# Evaluating Different Methods for Determining the Velocity-Dip Position over the Entire Cross Section and at the Centerline of a Rectangular Open Channel

**DOI:** 10.3390/e22060605

**Published:** 2020-05-28

**Authors:** Zhongfan Zhu, Pengfei Hei, Jie Dou, Dingzhi Peng

**Affiliations:** 1 Beijing Key Laboratory of Urban Hydrological Cycle and Sponge City Technology, College of Water Sciences, Beijing Normal University, Beijing 100875, China; zhuzhongfan1985@bnu.edu.cn; 2 College of Life and Environmental Science, Minzu University of China, Beijing 100081, China; heipf06@mails.tsinghua.edu.cn; 3 Department of Civil and Environmental Engineering, Nagaoka University of Technology, 1603-1, Kami-Tomioka, Nagaoka 940-2188, Japan; douj888@vos.nagaokaut.ac.jp

**Keywords:** probability method, Tsallis entropy, general index entropy, velocity-dip position, rectangular open channel

## Abstract

The velocity profile of an open channel is an important research topic in the context of open channel hydraulics; in particular, the velocity-dip position has drawn the attention of hydraulic scientists. In this study, analytical expressions for the velocity-dip position over the entire cross section and at the centerline of a rectangular open channel are derived by adopting probability methods based on the Tsallis and general index entropy theories. Two kinds of derived entropy-based expressions have the same mathematical form as a function of the lateral distance from the sidewall of the channel or of the aspect ratio of the channel. Furthermore, for the velocity-dip position over the entire cross section of the rectangular open channel, the derived expressions are compared with each other, as well as with two existing deterministic models and the existing Shannon entropy-based expression, using fifteen experimental datasets from the literature. An error analysis shows that the model of Yang et al. and the Tsallis entropy-based expression predict the lateral distribution of the velocity-dip position better than the other proposed models. For the velocity-dip position at the centerline of the rectangular open channel, six existing conventional models, the derived Tsallis and general index entropy-based expressions, and the existing Shannon entropy-based models are tested against twenty-one experimental datasets from the literature. The results show that the model of Kundu and the Shannon entropy-based expression have superior prediction accuracy with respect to experimental data compared with other models. With the exception of these models, the Tsallis entropy-based expression has the highest correlation coefficient value and the lowest root mean square error value for experimental data among the other models. This study indicates that the Tsallis entropy could be a good addition to existing deterministic models for predicting the lateral distribution of the velocity-dip position of rectangular open channel flow. This work also shows the potential of entropy-based expressions, the Shannon entropy and the Tsallis entropy in particular, to predict the velocity-dip position at the centerline of both narrow and wide rectangular open channels.

## 1. Introduction

**Figure 1:**
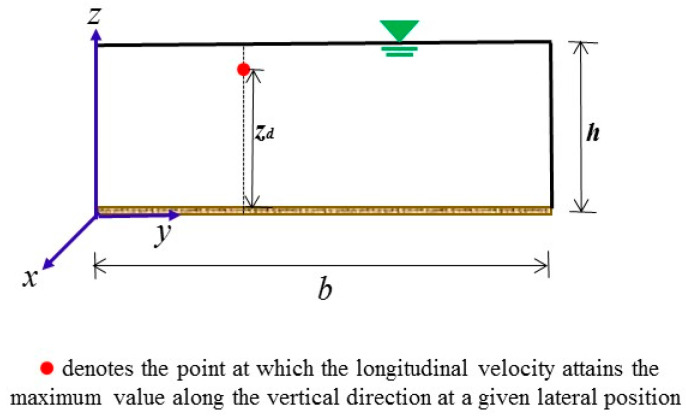
Velocity-dip position of rectangular open channel flow (redrawn from Kundu [9]). In this figure, the x-coordinate denotes the longitudinal direction, the y-coordinate represents the lateral direction and the z-coordinate represents the vertical direction; b and h denote the width and water depth of the open channel flow, respectively; and $z_{d}$ is the vertical distance of the position where the velocity-dip phenomenon occurs from the channel bottom.

The velocity distribution in an open channel turbulent flow plays an important role in evaluating the mean and maximum velocity, flow discharge, and shear stress across the channel and the sediment transport rate (e.g., [1,2,3]). Hydraulic engineers have carried out experiments to investigate the vertical distribution of longitudinal velocity in open channels (e.g., [2,4,5,6]). Some experiments have shown that the maximum velocity does not always appear at the water surface in open channels (e.g., [4,5,6,7,8]). The phenomenon of the maximum velocity happening below the water surface has been referred to as the velocity-dip phenomenon in the literature, and the maximum velocity position with respect to the bottom of the open channel has been referred to as the velocity-dip position (e.g., [2,5,6]), as schematically shown in Figure 1. This study only focuses on the rectangular open channel flows.

Many experiments have measured the lateral distribution of the velocity-dip position in open channels, including Yang [10] and NHRI [11]. Based on these experimental results, some empirical expressions have been proposed to characterize the location of maximum velocity of open channel flows for a given lateral distance from the sidewall of the channel [12]. For example, Wang et al. [13] proposed a linear expression including a sine function for the velocity-dip position, whereas Yang et al. [14] suggested a linear expression including an exponential function for the velocity-dip position. With regard to the velocity-dip position at the centerline of an open channel, some experiments have shown that the velocity-dip phenomenon happens when the aspect ratio of the channel $A_{r}$ (= b/h) is smaller than a certain value (e.g., [2,15,16]). Thus, the velocity-dip position at the centerline of an open channel is only influenced by the aspect ratio of open channel flow [2,15,16,17]. Some empirical models have been proposed to describe the relationship between the velocity-dip position at the centerline of the open channel and the aspect ratio of the channel, including those in the works of Wang et al. [13], Yang et al. [14], Absi [18], Bonakdari et al. [19], Guo [20], Pu [21] and Kundu [22].

These studies have provided effective methods for predicting the position of the maximum longitudinal velocity over the entire cross section and at the centerline of open channel flow. Recently, Kundu [9], Kundu [12], and Kundu and Ghoshal [23] adopted the probability method based on Shannon entropy to model the velocity-dip position across an entire section, as well as at the centerline of open channel flow. These works motivate us to explore the possibility of using two more general entropy theories, Tsallis entropy and general index entropy, to predict the velocity-dip position over the entire cross section and at the centerline of open channel flow. In fact, in recent years, Tsallis entropy has been widely applied to tackling some hydraulic engineering problems, such as the velocity profile prediction of open channel flow (e.g., [24,25]), the prediction of shear stress distribution in open channels (e.g., [26,27]), the estimation of suspended sediment concentration (e.g., [25,28]), the calculation of water distribution networks (e.g., [29]) and the flocculation dynamics modeling of cohesive sediment [30]. General index entropy has also been adopted to estimate the two-dimensional velocity distribution profile in open channel flow (e.g., [31]). Moreover, we find that no comprehensive and rigorous analysis has been performed to date that uses experimental data to compare the goodness of fit of these existing conventional deterministic methods and of entropy-based methods for predicting the location of the maximum longitudinal velocity over the entire cross section and at the centerline of open channel flow. Therefore, this study attempts to derive the analytical expression for the velocity-dip position over the entire cross section and at the centerline of open channel flow based on Tsallis entropy and general index entropy and to present a comparative study of existing conventional models and entropy-based expressions using experimental data from the literature.

This study is organized as follows. Section 2 summarizes the conventional models and derives the analytical expression based on different entropy theories for predicting the velocity-dip position over the entire cross section and at the centerline of open channel flow. A comparison among existing conventional models, three kinds of entropy-based expressions and experimental data are presented in Section 3, and Section 4 gives concluding remarks.

## 2. Conventional Model and Entropy-Based Expression for Determining the Velocity-Dip Position

### 2.1. Conventional Model for Velocity-Dip Position

Wang et al. [13] simply analyzed some experimental data regarding the location of maximum velocity in open channel flows and found that the following expression is in good agreement with the data:

(1) $$\xi_{d}=\frac{z_{d}}{h}=0.44+0.212\frac{y}{h}+0.05\sin\left(\frac{2\pi }{2.6}\frac{y}{h}\right)$$

where $\xi_{d}$ is the non-dimensional velocity-dip position for the whole cross section of the open channel: $\xi_{d}=\frac{z_{d}}{h}$, $z_{d}$ is the vertical distance of the velocity-dip position, h denotes the water depth of open channel flow, and y is the lateral distance, as shown in Figure 1. These experimental data originated from the works of Nezu and Rodi [32], Murphy [5], Gibson [33], Wang and Fu [34], Cardoso et al. [35], Song and Graf [36], Coleman [37], Wang and Qian [38] and Kironoto and Graf [39]. By observing the experimental data of Yang [10] and NHRI [11], Yang et al. [14] proposed another expression that also agrees well with the experimental data:

(2) $$\xi_{d}=\frac{1}{1+1.3\exp\left(-\frac{y}{h}\right)}$$

Regarding the velocity-dip position at the centerline of the open channel, Wang et al. [13] proposed the following empirical formula to estimate the elevation of the maximum velocity by evaluating the main factors affecting the velocity profile of open channel flows:

(3) $$\xi_{d}=0.44+0.106A_{r}+0.05\sin\left(\frac{\pi }{2.6}A_{r}\right)$$

Equation (3) was obtained by fitting it with collected experimental datasets, as already mentioned in Equation (1). In the work of Yang et al. [14], a dip-modified log law for the velocity distribution in smooth uniform open channel flows was proposed. According to this law, the velocity-dip position at the centerline of an open channel could be characterized by:

(4) $$\xi_{d}=\frac{1}{1+1.3\exp\left(-\frac{A_{r}}{2}\right)}$$

This expression has also been validated in the work of Absi [18]. Equation (4) was obtained by comparing it with the experimental data of Yang [10] and NHRI [11], as in Equation (2). By noticing both Equations (3) and (4) can present $\xi_{d}$ = 0.68 when $A_{r}$ is equal to 2, whereas the experimental results of Nezu and Rodi [15] showed a maximum longitudinal velocity at $\xi_{d}$ = 0.65, Bonakdari et al. [19] proposed a different sigmoid model for $\xi_{d}$ at the centerline of the open channel as follows:

(5) $$\xi_{d}=\frac{42.4+A_{r}{}^{4.2}}{94.7+A_{r}{}^{4.2}}$$

This equation was obtained by comparing with the experimental results of Nezu and Rodi [15], Wang and Qian [38], Cardoso et al. [35], Larrarte [40] and Tominaga et al. [41].

Furthermore, Guo [20] found that the velocity-dip position shifts exponentially from the free water surface to half the water depth as the aspect ratio of the open channel decreases from infinity to zero, and thus, put forward the following empirical formula to describe $\xi_{d}$, based on the experimental data of Hu and Hui [2]:

(6) $$\xi_{d}=\frac{1}{1+\exp\left[-{\left(\frac{A_{r}}{\pi }\right)}^{1.5}\right]}$$

As in the work of Bonakdari et al. [19], Pu [21] suggested the following velocity-dip position expression by comparing with the experimental results of Larrarte [40], Tominaga et al. [41] and Gordon [7]:

(7) $$\xi_{d}=\frac{40.1+A_{r}{}^{4.4}}{80.5+A_{r}{}^{4.4}}$$

Using the asymptotic matching technique, Kundu [22] proposed the following empirical model to predict the velocity-dip position at the centerline of open channel flow:

(8) $$\xi_{d}={\left[\frac{1}{\frac{1}{0.48^{16.09}+{\left(0.0895A_{r}+0.4718\right)}^{16.09}}+1}\right]}^{\frac{1}{16.09}}$$

which shows a good agreement with collected experimental data. These datasets will be presented in Section 3.1 of this paper.

It could be observed that these expressions were obtained empirically by the best-fitting method from different experimental datasets. Finally, note that they (Equations (1)–(7)) were first collected by Kundu [12] and Kundu [9]; we have presented them here in more detail in order to compare them with the entropy-based methods presented below.

### 2.2. Entropy-Based Expression for Velocity-Dip Position

In probability theory, the non-dimensional velocity-dip position $\xi_{d}$ should be considered a continuous random variable. To formulate the expression for the non-dimensional velocity-dip position by the entropy-based method, a hypothesis regarding the cumulative distribution function (CDF) of the non-dimensional velocity-dip position for the whole cross section and at the centerline of the open channel needs to be made.

The hypothesized CDF of the non-dimensional velocity-dip position for the entire cross section of the open channel should satisfy the following characteristics: it should be continuous and differentiable; it should change from zero to unity. Moreover, it should be able to reflect the characteristics of the velocity profile in open channel flows. To this end, Kundu and Ghoshal [23] proposed the following power law hypothesis for the CDF of the non-dimensional velocity-dip position $F\left(\xi_{d}\right)$:

(9) $$F\left(\xi_{d}\right)={\left(\frac{y}{y_{\max}}\right)}^{n}$$

which is in good agreement with the experimental data of Yang [10], NHRI [11] and Yan et al. [17] (the correlation coefficient reaches 0.98). In Equation (9), $y_{\max}$ is the maximum value of the lateral distance y, and n is the fitting value, which is larger than zero and characterizes the increasing pattern of the CDF. In general, the open rectangular channel flow is symmetrical with respect to the center line in a cross sectional plane. Thus, the maximum value of y could be considered to be half of the width [23], i.e., $y_{\max}$=b/2.

Similarly, the hypothesized CDF $F\left(\xi_{d}\right)$ of the non-dimensional velocity-dip position at the centerline of the open channel could reflect the characteristics of $\xi_{d}$ at the centerline of the open channel. Some previous studies have observed that the velocity-dip position $\xi_{d}$ in the middle of the open channel increases exponentially with the increasing aspect ratio of the open channel (e.g., [20]). Furthermore, Kundu [9] proposed the following type of CDF as a good choice:

(10) $$F\left(\xi_{d}\right)=1-\exp\left[-k_{1}{\left(A_{r}\right)}^{k_{2}}\right]$$

which is in good agreement with the experimental data points. In Equation (10), $k_{1}$ and $k_{2}$ are two fitting parameters that are larger than zero. Equation (10) is indeed a parametric expression with both $F\left(\xi_{d}\right)$ and $\xi_{d}$ given as functions of $A_{r}$. This expression was obtained by empirically fitting the calculated CDF values in the probability domain for collected experimental data. The mathematical form of this expression, which shows the monotonical increasing pattern of CDF to the unique variable (aspect ratio $A_{r}$), was determined based on collected experimental data, and was also consistent with the equations proposed by previous scholars (Equations (3)–(8)). More details regarding Equation (10) could be found in Kundu [9]. The hypothesized CDF (Equation (10)) has the following properties: it is differentiable and continuous, ranging from 0 to 1, and for infinitely narrow open channels ($A_{r}\to 0$), $F\left(\xi_{d}\right)$ approaches zero, whereas for infinitely wide open channels ($A_{r}\to \infty$), $F\left(\xi_{d}\right)$ reaches unity. By comparing Equation (10) with collected experimental data points, Kundu [9] found that the values of $k_{1}$ and $k_{2}$ are 0.0698 and 1.8767, respectively.

The determination of the velocity-dip position over the entire cross section and at the centerline of the open channel by the application of entropy theory follows the procedures shown in a series of previous entropy-based works (e.g., [42,43]): (1) Definition of the continuous form of the entropy function; (2) Specification of the constraint conditions to which the velocity-dip position is subjected; (3) Maximization of the entropy function and derivation of the probability density function (PDF) of the velocity-dip position; (4) Specification and estimation of Lagrange multipliers; and (5) Derivation of the velocity-dip position.

#### 2.2.1. Tsallis Entropy for the Velocity-Dip Position

Tsallis [44] put forward a generalized form of informational entropy; its continuous form of $\xi_{d}$, denoted $H_{T}\left(\xi_{d}\right)$, can be written as:

(11) $$H_{T}\left(\xi_{d}\right)=\frac{1}{m-1}\int_{\xi_{d\min}}^{\xi_{d\max}}f\left(\xi_{d}\right)\left\{1-{\left[f\left(\xi_{d}\right)\right]}^{m-1}\right\}d\xi_{d}$$

where m is the Tsallis entropy index (which is a real number not equal to unity), $\xi_{d\min}$ and $\xi_{d\max}$ are the minimum and maximum values of $\xi_{d}$, respectively, $f\left(\xi_{d}\right)$ is the PDF of $\xi_{d}$, and $H_{T}\left(\xi_{d}\right)$ is the Tsallis entropy function of $\xi_{d}$. Equation (11) expresses a measure of the uncertainty associated with $f\left(\xi_{d}\right)$ or $\xi_{d}$.

Two constraint conditions on the PDF of $\xi_{d}$ are:

(12) $$\int_{\xi_{d\min}}^{\xi_{d\max}}f\left(\xi_{d}\right)=1$$

(13) $$\int_{\xi_{d\min}}^{\xi_{d\max}}\xi_{d}f\left(\xi_{d}\right)=\bar{\xi_{d}}$$

where $\bar{\xi_{d}}$ is the average (or mean) value of $\xi_{d}$ determined from the observation data. Equation (12) is derived from the definition of the PDF, and Equation (13) presents the mean (or average) constraint condition.

To choose among all PDFs satisfying Equations (12) and (13), the principle of maximum entropy developed by Jaynes [45,46,47] is adopted in this study. The maximum entropy principle states that the PDF should be selected in a way that maximizes the entropy function while being subject to constraint equations. One common method of maximizing the entropy function is to adopt the method of the Euler–Lagrange calculus of variations [25,42]. Therefore, the Lagrange function $L_{T}\left(\xi_{d}\right)$ can be constructed as follows:

(14) $$\begin{array}{l} L_{T}\left(\xi_{d}\right)=\frac{1}{m-1}\left\{\int_{\xi_{d\min}}^{\xi_{d\max}}f\left(\xi_{d}\right)d\xi_{d}-\int_{\xi_{d\min}}^{\xi_{d\max}}{\left[f\left(\xi_{d}\right)\right]}^{m}d\xi_{d}\right\}\\ -\lambda_{T1}\left[\int_{\xi_{d\min}}^{\xi_{d\max}}f\left(\xi_{d}\right)d\xi_{d}-1\right]-\lambda_{T2}\left[\int_{\xi_{d\min}}^{\xi_{d\max}}\zeta f\left(\xi_{d}\right)d\xi_{d}-\bar{\xi_{d}}\right] \end{array}$$

where $\lambda_{T1}$ and $\lambda_{T2}$ are two Lagrange multipliers for the Tsallis entropy.

Differentiating Equation (14) with respect to $f\left(\xi_{d}\right)$ (here $f\left(\xi_{d}\right)$ is taken as the variable and $\xi_{d}$ as a parameter) and setting this derivative to be zero could result in the least-biased PDF of $\xi_{d}$, as follows:

(15) $$f\left(\xi_{d}\right)={\left[\frac{m-1}{m}\left(\frac{1}{m-1}-\lambda_{T1}-\lambda_{T2}\xi_{d}\right)\right]}^{\frac{1}{m-1}}$$

Furthermore, integrating Equation (15) from $\xi_{d\min}$ to $\xi_{d}$ can lead to the CDF of $\xi_{d}$, $F\left(\xi_{d}\right)$, as follows:

(16) $$F\left(\xi_{d}\right)={\left(\frac{m-1}{m}\right)}^{\frac{m}{m-1}}\frac{1}{\lambda_{T2}}\left[{\left(\frac{1}{m-1}-\lambda_{T1}-\lambda_{T2}\xi_{d\min}\right)}^{\frac{m}{m-1}}-{\left(\frac{1}{m-1}-\lambda_{T1}-\lambda_{T2}\xi_{d}\right)}^{\frac{m}{m-1}}\right]$$

and substituting Equation (15) into Equation (11) can lead to the maximum Tsallis entropy function $H_{T}\left(\xi_{d}\right)$, as follows:

(17) $$H_{T}\left(\xi_{d}\right)=\frac{1}{m-1}\left\{\begin{array}{l} \left(\xi_{d\max}-\xi_{d\min}\right)+{\left(\frac{m-1}{m}\right)}^{\frac{m}{m-1}}\frac{1}{\left(2m-1\right)}\frac{1}{\lambda_{T2}}\\ {}*\left[{\left(\frac{1}{m-1}-\lambda_{T1}-\lambda_{T2}\xi_{d\max}\right)}^{\frac{2m-1}{m-1}}-{\left(\frac{1}{m-1}-\lambda_{T1}-\lambda_{T2}\xi_{d\min}\right)}^{\frac{2m-1}{m-1}}\right] \end{array}\right\}$$

Finally, substituting Equation (15) into the two constraint equations (Equations (12) and (13)) can yield:

(18) $$\frac{1}{\lambda_{T2}}{\left(\frac{m-1}{m}\right)}^{\frac{m}{m-1}}\left[{\left(\frac{1}{m-1}-\lambda_{T1}-\lambda_{T2}\xi_{d\min}\right)}^{\frac{m}{m-1}}-{\left(\frac{1}{m-1}-\lambda_{T1}-\lambda_{T2}\xi_{d\max}\right)}^{\frac{m}{m-1}}\right]=1$$

(19) $$\begin{array}{l} \xi_{d\max}{\left(\frac{1}{m-1}-\lambda_{T1}-\lambda_{T2}\xi_{d\max}\right)}^{\frac{m}{m-1}}-\xi_{d\min}{\left(\frac{1}{m-1}-\lambda_{T1}-\lambda_{T2}\xi_{d\min}\right)}^{\frac{m}{m-1}}\\ +\frac{m-1}{2m-1}\frac{1}{\lambda_{T2}}\left[{\left(\frac{1}{m-1}-\lambda_{T1}-\lambda_{T2}\xi_{d\max}\right)}^{\frac{2m-1}{m-1}}-{\left(\frac{1}{m-1}-\lambda_{T1}-\lambda_{T2}\xi_{d\min}\right)}^{\frac{2m-1}{m-1}}\right]\\ +\lambda_{T2}\bar{\xi_{d}}{\left(\frac{m}{m-1}\right)}^{\frac{m}{m-1}}=0 \end{array}$$

Equations (18) and (19) constitute a non-linear equation system, which can be solved to determine the two Lagrange multipliers, $\lambda_{T1}$ and $\lambda_{T2}$, for known values of $\xi_{d\min}$, $\xi_{d\max}$ and $\bar{\xi_{d}}$ as well as the Tsallis entropy index m.

Combining Equations (9), (16) and (18) can give the following expression for the non-dimensional velocity-dip position $\xi_{d}$ for the whole cross section of an open channel based on the Tsallis entropy:

(20) $$\begin{array}{l} \xi_{d}=-\frac{1}{\lambda_{T2}}{\left\{\begin{array}{l} {\left(\frac{1}{m-1}-\lambda_{T1}-\lambda_{T2}\xi_{d\min}\right)}^{\frac{m}{m-1}}+\left[\begin{array}{l} {\left(\frac{1}{m-1}-\lambda_{T1}-\lambda_{T2}\xi_{d\max}\right)}^{\frac{m}{m-1}}\\ -{\left(\frac{1}{m-1}-\lambda_{T1}-\lambda_{T2}\xi_{d\min}\right)}^{\frac{m}{m-1}} \end{array}\right]\\ {}*{\left(\frac{y}{\frac{1}{2}b}\right)}^{n} \end{array}\right\}}^{\frac{m-1}{m}}\\ -\frac{\lambda_{T1}}{\lambda_{T2}}+\frac{1}{\lambda_{T2}\left(m-1\right)} \end{array}$$

By equating Equations (10), (16) and (18), the dimensionless velocity-dip position $\xi_{d}$ at the centerline of the open channel can be expressed as:

(21) $$\begin{array}{l} \xi_{d}=-\frac{1}{\lambda_{T2}}{\left\{\begin{array}{l} {\left(\frac{1}{m-1}-\lambda_{T1}-\lambda_{T2}\xi_{d\max}\right)}^{\frac{m}{m-1}}-\left[\begin{array}{l} {\left(\frac{1}{m-1}-\lambda_{T1}-\lambda_{T2}\xi_{d\max}\right)}^{\frac{m}{m-1}}\\ -{\left(\frac{1}{m-1}-\lambda_{T1}-\lambda_{T2}\xi_{d\min}\right)}^{\frac{m}{m-1}} \end{array}\right]\\ {}*\exp\left[-k_{1}{\left(A_{r}\right)}^{k_{2}}\right] \end{array}\right\}}^{\frac{m-1}{m}}\\ -\frac{\lambda_{T1}}{\lambda_{T2}}+\frac{1}{\lambda_{T2}\left(m-1\right)} \end{array}$$

#### 2.2.2. General Index Entropy for the Velocity-Dip Position

Shorrocks [48] presented the general index entropy based on a random variable and an entropy index α; the continuous form of this kind of entropy function for $\xi_{d}$, $H_{G}\left(\xi_{d}\right)$, can be expressed as:

(22) $$H_{G}(\xi_{d})=\frac{1}{\alpha \left(\alpha -1\right)}\int_{\xi_{d\min}}^{\xi_{d\max}}{\left[f\left(\xi_{d}\right)\right]}^{\alpha }d\xi_{d}$$

where α is the general index entropy index as defined above. Here, in general, α is greater than zero and is not equal to unity, whereas as α becomes close to 1, the general index entropy reduces to the Shannon entropy.

The two constraint conditions are still Equations (12) and (13). To derive the least-biased PDF of $\xi_{d}$ from the principle of maximum entropy, the Lagrange function $L_{G}\left(\xi_{d}\right)$ for the general index entropy can be constructed as follows:

(23) $$L_{G}\left(\xi_{d}\right)=\frac{1}{\alpha \left(\alpha -1\right)}{\left[f\left(\xi_{d}\right)\right]}^{\alpha }+\lambda_{G1}f\left(\xi_{d}\right)+\lambda_{G2}\xi_{d}f\left(\xi_{d}\right)$$

where $\lambda_{G1}$ and $\lambda_{G2}$ are two Lagrange multipliers for the general index entropy.

Taking the derivative of Equation (23) with respect to $f\left(\xi_{d}\right)$ and setting it equal to zero yields the following expression for the PDF of $\xi_{d}$, $f\left(\xi_{d}\right)$:

(24) $$f(\xi_{d})={\left(1-\alpha \right)}^{\frac{1}{\alpha -1}}{\left(\lambda_{G1}+\lambda_{G2}\xi_{d}\right)}^{\frac{1}{\alpha -1}}$$

By integrating Equation (24) from $\xi_{d\min}$ to $\xi_{d}$, the CDF of $\xi_{d}$ is determined to be:

(25) $$F\left(\xi_{d}\right)=\frac{1}{\lambda_{G2}\alpha }{\left(1-\alpha \right)}^{\frac{\alpha }{\alpha -1}}\left[\begin{array}{l} {\left(\lambda_{G1}+\lambda_{G2}\xi_{d\min}\right)}^{\frac{\alpha }{\alpha -1}}\\ -{\left(\lambda_{G1}+\lambda_{G2}\xi_{d}\right)}^{\frac{\alpha }{\alpha -1}} \end{array}\right]$$

and substituting Equation (24) into Equation (22) yields the maximum $H_{G}(\xi_{d})$, as follows:

(26) $$\begin{array}{l} H_{G}(\xi_{d})=\frac{1}{\alpha \left(\alpha -1\right)}\int_{\xi_{d}{}_{\min}}^{\xi_{d}{}_{\max}}{\left(\frac{\lambda_{G1}+\lambda_{G2}\xi_{d}}{1-\alpha_{R}}\right)}^{\frac{\alpha }{\alpha -1}}d\xi_{d}\\ =\frac{1}{\lambda_{G2}\alpha \left(2\alpha -1\right)}{\left(1-\alpha \right)}^{\frac{\alpha }{1-\alpha }}\left[{\left(\lambda_{G1}+\lambda_{G2}\xi_{d\max}\right)}^{\frac{2\alpha -1}{\alpha -1}}-{\left(\lambda_{G1}+\lambda_{G2}\xi_{d\min}\right)}^{\frac{2\alpha -1}{\alpha -1}}\right] \end{array}$$

Similarly, the non-linear equation system for the determination of the two Lagrange multipliers $\lambda_{G1}$ and $\lambda_{G2}$ can be obtained by inserting Equation (24) into the two constraint equations, Equations (12) and (13), as follows:

(27) $$\frac{1}{\lambda_{G2}\alpha }{\left(1-\alpha \right)}^{\frac{\alpha }{\alpha -1}}\left[\begin{array}{l} {\left(\lambda_{G1}+\lambda_{G2}\xi_{d\min}\right)}^{\frac{\alpha }{\alpha -1}}\\ -{\left(\lambda_{G1}+\lambda_{G2}\xi_{d\max}\right)}^{\frac{\alpha }{\alpha -1}} \end{array}\right]-1=0$$

(28) $$\begin{array}{l} \frac{\left(\alpha -1\right)}{\lambda_{G2}\alpha }{\left(1-\alpha \right)}^{\frac{1}{\alpha -1}}\left\{\begin{array}{l} \xi_{d\max}{\left(\lambda_{G1}+\lambda_{G2}\xi_{d\max}\right)}^{\frac{\alpha }{\alpha -1}}-\xi_{d\min}{\left(\lambda_{G1}+\lambda_{G2}\xi_{d\min}\right)}^{\frac{\alpha }{\alpha -1}}\\ -\frac{1}{\lambda_{G2}}\frac{\left(\alpha -1\right)}{\left(2\alpha -1\right)}*\\ \left[{\left(\lambda_{G1}+\lambda_{G2}\xi_{d\max}\right)}^{\frac{2\alpha -1}{\alpha -1}}-{\left(\lambda_{G1}+\lambda_{G2}\xi_{d\min}\right)}^{\frac{2\alpha -1}{\alpha -1}}\right] \end{array}\right\}\\ -\bar{\xi_{d}}=0 \end{array}$$

Finally, the non-dimensional velocity-dip position $\xi_{d}$ over the entire cross section of open channel flow can be obtained based on the general index entropy by combining Equations (9), (25) and (27):

(29) $$\xi_{d}=-\frac{\lambda_{G1}}{\lambda_{G2}}+\frac{1}{\lambda_{G2}}{\left\{\begin{array}{l} {\left(\lambda_{G1}+\lambda_{G2}\xi_{d\min}\right)}^{\frac{\alpha }{\alpha -1}}+\left[\begin{array}{l} {\left(\lambda_{G1}+\lambda_{G2}\zeta_{\max}\right)}^{\frac{\alpha }{\alpha -1}}\\ -{\left(\lambda_{G1}+\lambda_{G2}\zeta_{\min}\right)}^{\frac{\alpha }{\alpha -1}} \end{array}\right]\\ {}*{\left(\frac{y}{\frac{1}{2}b}\right)}^{n} \end{array}\right\}}^{\frac{\alpha -1}{\alpha }}$$

Combining Equations (10), (25) and (27) yields the expression for the non-dimensional general index entropy-based velocity-dip position $\xi_{d}$ at the centerline of the open channel, as follows:

(30) $$\xi_{d}=-\frac{\lambda_{G1}}{\lambda_{G2}}+\frac{1}{\lambda_{G2}}{\left\{\begin{array}{l} {\left(\lambda_{G1}+\lambda_{G2}\zeta_{d}{}_{\max}\right)}^{\frac{\alpha }{\alpha -1}}-\left[\begin{array}{l} {\left(\lambda_{G1}+\lambda_{G2}\xi_{d}{}_{\max}\right)}^{\frac{\alpha }{\alpha -1}}\\ -{\left(\lambda_{G1}+\lambda_{G2}\xi_{d}{}_{\min}\right)}^{\frac{\alpha }{\alpha -1}} \end{array}\right]\\ {}*\exp\left[-k_{1}{\left(A_{r}\right)}^{k_{2}}\right] \end{array}\right\}}^{\frac{\alpha -1}{\alpha }}$$

#### 2.2.3. Shannon Entropy for the Velocity-Dip Position

The Shannon entropy function for the non-dimensional velocity-dip position $\xi_{d}$, $H_{S}\left(\xi_{d}\right)$, can be expressed as [49]:

(31) $$H_{S}(\xi_{d})=-\int_{\xi_{d\min}}^{\xi_{d\max}}f\left(\xi_{d}\right)\ln\left[f\left(\xi_{d}\right)\right]d\xi_{d}$$

Following the aforementioned procedures, Kundu and Ghoshal [23] derived the Shannon entropy-based expression for the velocity-dip position over the whole cross section of the open channel. Here, we reorganize the Shannon entropy-based expression for the non-dimensional velocity-dip position $\xi_{d}$ in the following form:

(32) $$\xi_{d}=\frac{1}{\lambda_{S2}}\ln\left\{\exp\left(\lambda_{S2}\xi_{d\min}\right)+\left[\exp\left(\lambda_{S2}\xi_{d\max}\right)-\exp\left(\lambda_{S2}\xi_{d\min}\right)\right]{\left(\frac{y}{\frac{1}{2}b}\right)}^{n}\right\}$$

where $\lambda_{S2}$ is one of the Lagrange multipliers for the Shannon entropy. The values of $\lambda_{S2}$, as well as of the other Lagrange multiplier $\lambda_{S1}$, can be determined by solving the following non-linear equation system:

(33) $$\frac{\exp\left(\lambda_{S1}-1\right)}{\lambda_{S2}}\left[\exp\left(\lambda_{S2}\xi_{d\max}\right)-\exp\left(\lambda_{S2}\xi_{d\min}\right)\right]=1$$

(34) $$\begin{array}{l} \frac{\exp\left(\lambda_{S1}-1\right)}{\lambda_{S2}^{2}}\left[\exp\left(\lambda_{S2}\xi_{d\max}\right)\left(\lambda_{S2}\xi_{d\max}-1\right)-\exp\left(\lambda_{S2}\xi_{d\min}\right)\left(\lambda_{S2}\xi_{d\min}-1\right)\right]\\ =\bar{\xi_{d}} \end{array}$$

By adopting the Shannon entropy theory, Kundu [9] derived the expression for the non-dimensional velocity-dip position $\xi_{d}$ at the centerline of the open channel as follows:

(35) $$\xi_{d}=\xi_{d\min}+\frac{1}{2M_{S}}\ln\left\{1+\left[\exp\left(M_{S}\right)-1\right]\left[1-\exp(-k_{1}{\left(A_{r}\right)}^{k_{2}})\right]\right\}$$

where $M_{S}$ is an entropy parameter defined by Kundu [9] as follows: $M_{S}=\left(\xi_{d\max}-\xi_{d\min}\right)\lambda_{S2}$.

#### 2.2.4. Reparameterization of Two Kinds of Entropy-Based Models

Introducing a new Tsallis entropy parameter $P_{T}=\left(\frac{1}{m-1}-\lambda_{T1}\right)/\lambda_{T2}$ into Equation (20) yields the following expression for the non-dimensional velocity-dip position $\xi_{d}$ for the whole cross section of the open channel, based on the Tsallis entropy:

(36) $$\xi_{d}=P_{T}-{\left\{{\left(P_{T}-\xi_{d\min}\right)}^{\frac{m}{m-1}}+\left[{\left(P_{T}-\xi_{d\max}\right)}^{\frac{m}{m-1}}-{\left(P_{T}-\xi_{d\min}\right)}^{\frac{m}{m-1}}\right]{\left(\frac{y}{\frac{1}{2}b}\right)}^{n}\right\}}^{\frac{m-1}{m}}$$

The expression of the Tsallis entropy-based dimensionless velocity-dip position $\xi_{d}$ at the centerline of the open channel (Equation (21)) becomes:

(37) $$\xi_{d}=P_{T}-{\left\{\begin{array}{l} {\left(P_{T}-\xi_{d\max}\right)}^{\frac{m}{m-1}}-\left[{\left(P_{T}-\xi_{d\max}\right)}^{\frac{m}{m-1}}-{\left(P_{T}-\xi_{d\min}\right)}^{\frac{m}{m-1}}\right]\\ {}*\exp\left[-k_{1}*{\left(A_{r}\right)}^{k_{2}}\right] \end{array}\right\}}^{\frac{m-1}{m}}$$

Similarly, introducing a new general index entropy parameter $P_{G}=-\lambda_{G1}/\lambda_{G2}$ into Equation (29) leads to the general index entropy-based velocity-dip position expression $\xi_{d}$ for the entire cross section of the open channel:

(38) $$\xi_{d}=P_{G}-{\left\{{\left(P_{G}-\xi_{d\min}\right)}^{\frac{\alpha }{\alpha -1}}+\left[{\left(P_{G}-\xi_{d\max}\right)}^{\frac{\alpha }{\alpha -1}}-{\left(P_{G}-\xi_{d\min}\right)}^{\frac{\alpha }{\alpha -1}}\right]{\left(\frac{y}{\frac{1}{2}b}\right)}^{n}\right\}}^{\frac{\alpha -1}{\alpha }}$$

In addition, Equation (30) becomes:

(39) $$\xi_{d}=P_{G}-{\left\{\begin{array}{l} {\left(P_{G}-\xi_{d\max}\right)}^{\frac{\alpha }{\alpha -1}}-\left[{\left(P_{G}-\xi_{d\max}\right)}^{\frac{\alpha }{\alpha -1}}-{\left(P_{G}-\xi_{d\min}\right)}^{\frac{\alpha }{\alpha -1}}\right]\\ {}*\exp\left[-k_{1}*{\left(A_{r}\right)}^{k_{2}}\right] \end{array}\right\}}^{\frac{\alpha -1}{\alpha }}$$

There are also some studies to define the dimensionless entropy parameter in order to reparameterize the entropy-based expressions (e.g., [50]). It can be observed that the Tsallis and general index entropy-based expressions (Equations (36) and (38)) for the velocity-dip position over the entire cross section of the open channel have the same mathematical form; the only difference lies in the different forms of the defined entropy parameter and the entropy index. It also needs to be pointed out that the Tsallis entropy parameter and general index entropy parameter ($P_{T}$ and $P_{G}$) contain Lagrange multipliers which are particular to the constraints, not containing a conceptual interpretation, and they are only defined in this way such that the mathematical forms of the entropy-based velocity-dip position expressions (Equations (20) and (21) and Equations (29) and (30)) can be greatly simplified for later calculations in this study.

**Figure 2:**
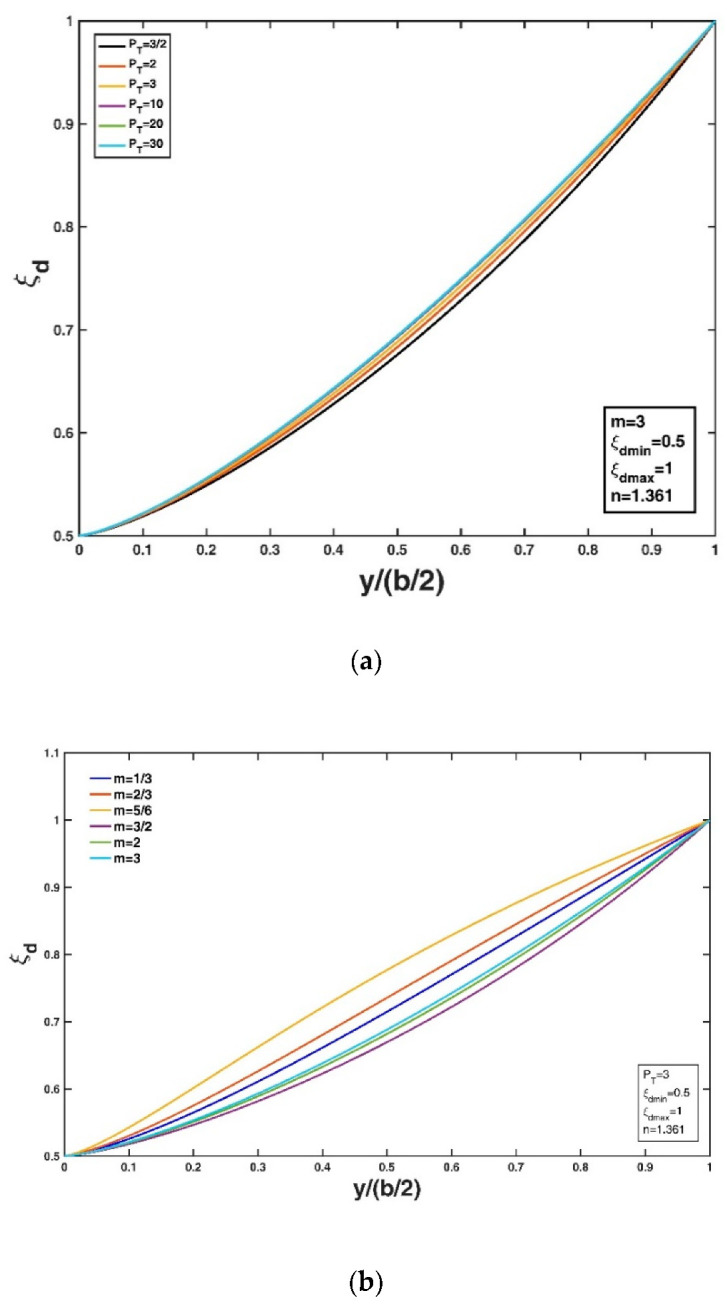
Variation in the non-dimensional velocity-dip position $\xi_{d}$ over the entire cross section of the open channel relative to the Tsallis entropy parameter $P_{T}$ (**a**), and the Tsallis entropy index m (**b**).

**Figure 3:**
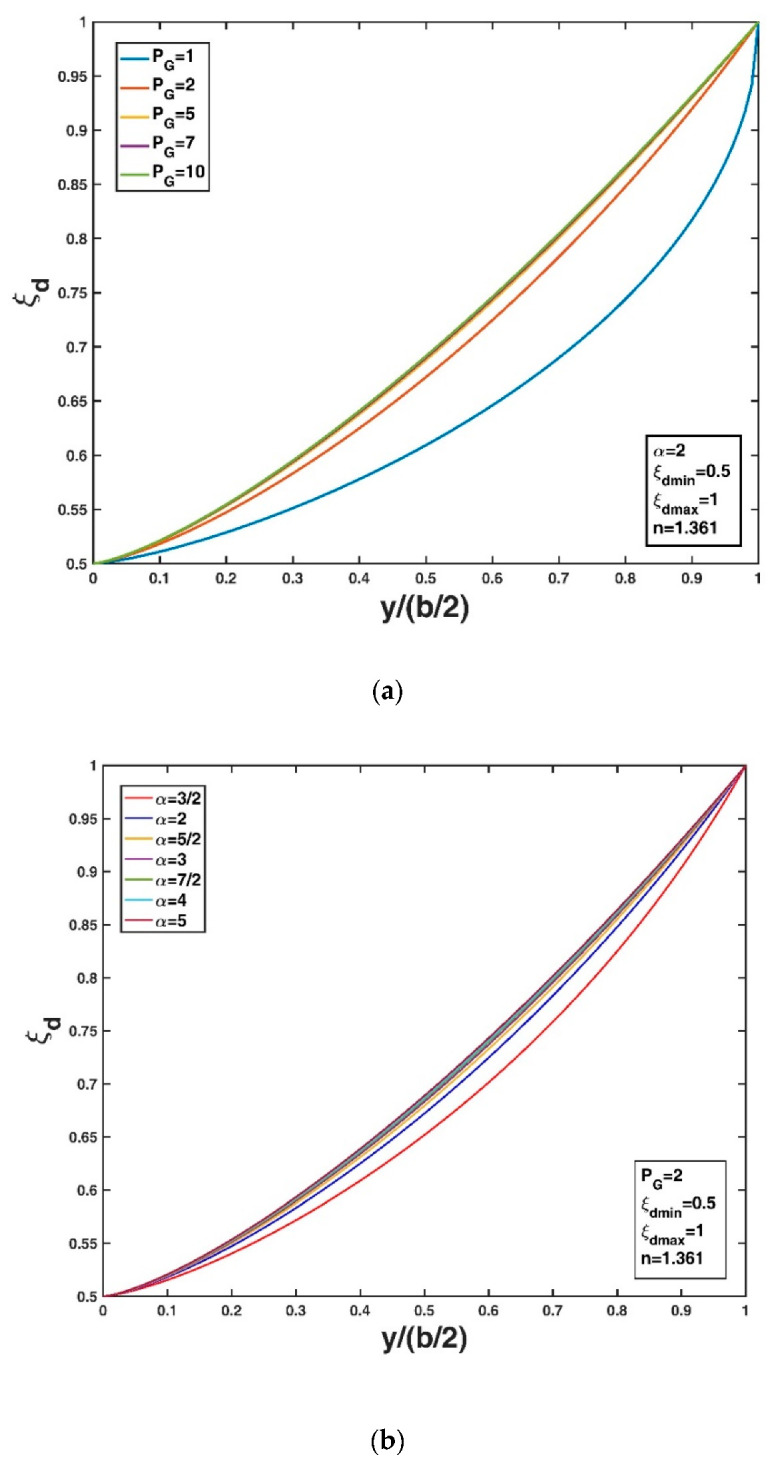
Variation in the non-dimensional velocity-dip position $\xi_{d}$ over the entire cross section of the open channel relative to the general index entropy parameter $P_{G}$ (**a**), and the general index entropy index α (**b**).

Figure 2a,b show the variation in the non-dimensional Tsallis entropy-based velocity-dip position relative to the new Tsallis entropy parameter and Tsallis entropy index, respectively, for fixed values of $\xi_{d\min}$ = 0.5 and $\xi_{d\max}$ = 1 (assuming a very wide or very shallow open rectangular channel) and n = 1.361 as given in Kundu and Ghoshal [23] as a typical example. Here, m = 3 is adopted, as by Cui and Singh [24,25] and Singh and Cui [50]. From Figure 2a, it can be seen that the velocity-dip position increases at a fixed lateral distance from the sidewall of the open channel as $P_{T}$ increases, while the lateral distribution of the velocity-dip position becomes insensitive to the variation in $P_{T}$ when $P_{T}$ exceeds 10. Figure 2b shows that the velocity-dip position increases at a fixed lateral distance y/h as m increases within the range 0<m<1 or within the range m>1. This complicated dependence of the velocity-dip position distribution on the entropy index m results from the fact that the entropy parameter $P_{T}$ is simply regarded to be a constant in Figure 2b. However, for the experimental datasets, since the entropy parameter is not independent of the entropy index, the entropy parameter $P_{T}$ could be determined as long as the Lagrange multipliers are obtained by using the non-linear equation system (Equations (18) and (19)) for a given entropy index m. Fixing the values of $\xi_{d\min}$ = 0.5, $\xi_{d\max}$ = 1 (assuming a very wide or very shallow open rectangular channel) and n = 1.361 as in Kundu and Ghoshal [23], Figure 3a,b show the variation in the velocity-dip-position relative to the new general index entropy parameter and the general index entropy index, respectively. Here, α = 2 is adopted as an example. The velocity-dip position increases greatly at a fixed lateral distance in the open channel as $P_{G}$ increases, but it becomes insensitive to the variation in $P_{G}$ when $P_{G}$ exceeds 5 (Figure 3a). For a given $P_{G}$ value, the velocity-dip position improves at a fixed lateral distance y/h as α increases from 3/2 to 5/2; however, a continual increase in α when α > 5/2 has a minor impact on the lateral distribution of the velocity-dip position over the entire cross section of the open channel (Figure 3b).

**Figure 4:**
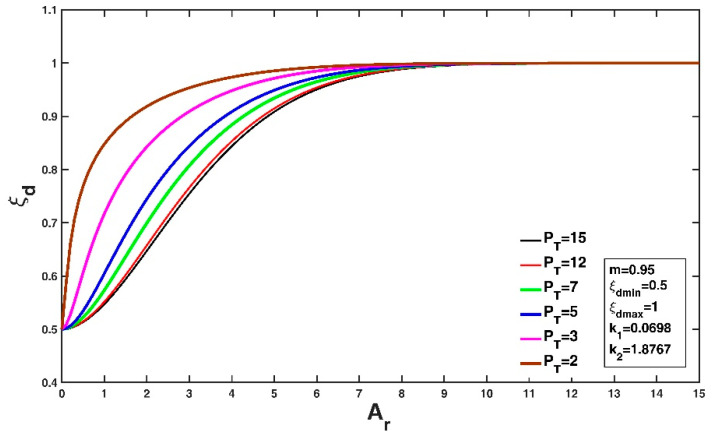
Variation of the velocity-dip position with the dimensionless entropy parameter for the Tsallis entropy-based expression (Equation (37)) with a fixed value of the entropy index (m = 0.95)

**Figure 5:**
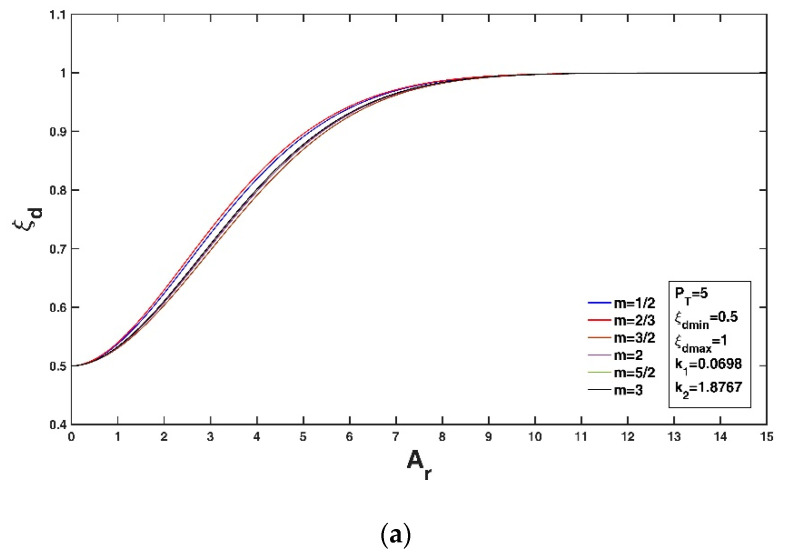

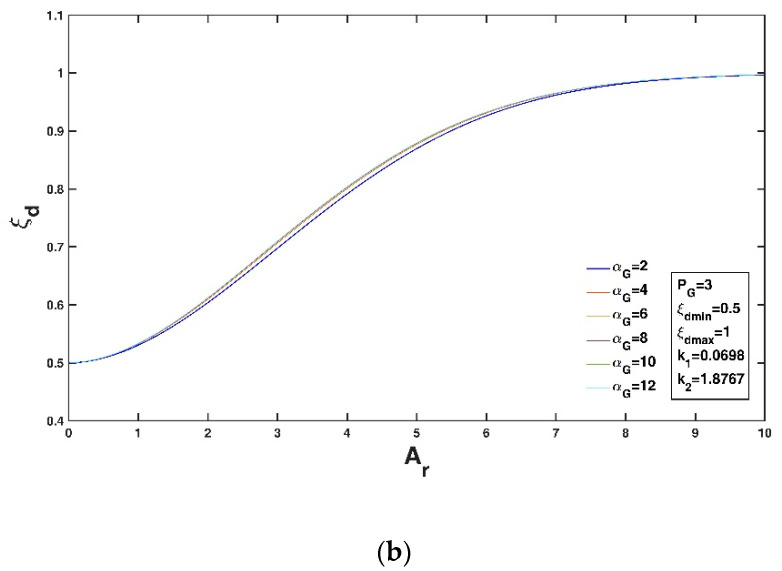
Variation of the velocity-dip position with the entropy index at fixed values of the entropy parameters: $P_{T}$ = 5 for the Tsallis entropy (**a**), and $P_{G}$ = 3 for the general index entropy (**b**).

Similarly, both the Tsallis entropy and the general index entropy yield expressions for the velocity-dip position at the centerline of the open channel with the same mathematical form (Equations (37) and (39)) but with different forms for the defined entropy parameter and entropy index. Taking Equation (37) with $\xi_{d\min}$ = 0.5 (implying a very narrow or very deep open rectangular channel, as shown in the experiment of Hu and Hui [2]) and $\xi_{d\max}$ = 1 (implying a very wide or very shallow open rectangular channel, as presented in the works of Hu and Hui [2], Nezu and Rodi [15], Guo and Julien [16] and Guo [20] as adopted by Kundu [9]) as an example and using $k_{1}$ = 0.0698 and $k_{2}$ = 1.8767, Figure 4 shows the variation in the velocity-dip position relative to the dimensionless entropy parameter at a fixed value of the entropy index m = 0.95. It can be observed that the increase in $P_{T}$ does not change the pattern of the $\xi_{d}$-$A_{r}$ relationship, and for a given open channel (fixed $A_{r}$), a large $P_{T}$ leads to a higher velocity-dip position when $0<A_{r}<8$. The variation becomes insignificant with increasing $P_{T}$ when $P_{T}$ exceeds 12. The general index entropy-based expression (Equation (39)) has similar characteristics. The impact of the entropy index on the variation in the velocity-dip position at a fixed entropy parameter for the Tsallis entropy and for the general index entropy is presented in Figure 5a,b, respectively. The increasing m slightly affects the variation in the velocity-dip position (Figure 5a), while the velocity-dip position is less sensitive to the change in the general index entropy index α (Figure 5b).

## 3. Comparison with Experimental Data and Discussion

### 3.1. Collected Experimental Datasets

Fifteen experimental datasets collected by Kundu [12] regarding the velocity-dip position across the entire cross section of the open channel are adopted to test the validity of conventional and entropy-based methods, including those in Yang [10] and NHRI [11]. In the experiment of Yang [10], the lateral vertical profile of longitudinal velocity in a rectangular open channel was measured. The length, width and depth of the open channel were 20 m, 49.2 cm, and 45 cm, respectively, and the aspect ratio of the open channel varied from 4.47 to 9.84. There were 88 collected experimental data points. A 120 cm wide open channel flow experiment was conducted in NHRI [11]. In this series of experiments, the aspect ratio of the open channel varied from 4.1 to 15, with a total of 24 experimental data points. It can be seen that collected experimental datasets cover a wide range of aspect ratios of the open channel, ranging from 4.1 to 15, which is reasonable for testing the validity of all of the proposed models.

**Figure 6:**
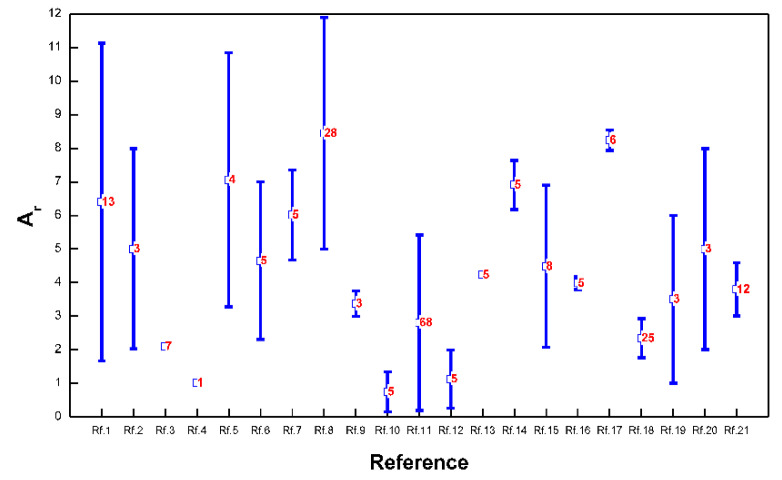
Information concerning twenty-one experimental datasets collected from the literature by Kundu [9]. The horizontal axis denotes the references: here, Rf.1 corresponds to Hu [51]; Rf.2: Sarma et al. [52]; Rf.3: Coleman [37]; Rf.4: Knight and Macdonald [53]; Rf.5: Rajaratnam and Muralidhar [54]; Rf.6: Yan et al. [17]; Rf.7: Cardoso et al. [35]; Rf.8: Vanoni [6]; Rf.9: Wang and Qian [38]; Rf.10: Murphy [5]; Rf.11: Nezu and Rodi [32]; Rf.12: Gibson [33]; Rf.13: Wang and Fu [34]; Rf.14: Zippe and Graf [55]; Rf.15: Kironoto and Graf [39]; Rf.16: Wang and An [56]; Rf.17: Guy et al. [57]; Rf.18: Larrarte [40]; Rf.19: Nezu and Rodi [15]; Rf.20: Tominaga et al. [41]; and Rf.21: Song and Graf [36]. The vertical axis denotes the aspect ratio of the open channel adopted in each experiment by each reference. The blue bars represent the range of aspect ratios adopted in the open channel experiments, and the red number in the middle of each bar shows the number of experimental data points.

For the velocity-dip position at the centerline of the open channel, twenty-one experimental datasets of open rectangular channels collected by Kundu [9] from the literature are presented in this study. These experimental datasets include those from Hu [51], Sarma et al. [52], Coleman [37], Knight and Macdonald [53], Rajaratnam and Muralidhar [54], Yan et al. [17], Cardoso et al. [35], Vanoni [6], Wang and Qian [38], Murphy [5], Nezu and Rodi [32], Gibson [33], Wang and Fu [34], Zippe and Graf [55], Kironoto and Graf [39], Wang and An [56], Guy et al. [57], Larrarte [40], Nezu and Rodi [15], Tominaga et al. [41] and Song and Graf [36]. Figure 6 presents information concerning these datasets, including the range of aspect ratios of the open channel adopted in this experiment by each reference (as shown by the blue bars in this figure) and the number of data points for each reference (as shown by the red numbers in this figure). It can be seen that the aspect ratio of the open channel has a wide range from 0.1552 to 11.8951 in the literature, and the total number of collected data points reaches 219.

### 3.2. Error Estimation

To evaluate the accuracy of the proposed models and other models with regard to the experimental data points, an error analysis is carried out in this study. In the error analysis, four statistical parameters are calculated for each case, which are computed as follows:

(1) The correlation coefficient *R*^2^ between the observed data points and the modeled data points:
  (40) $$R^{2}=\frac{{\left[\sum_{i=1}^{N}\left(\xi_{Oi}-\bar{\xi_{O}}\right)\left(\xi_{Mi}-\bar{\xi_{M}}\right)\right]}^{2}}{{\sum_{i=1}^{N}\left(\xi_{Oi}-\bar{\xi_{O}}\right)}^{2}{\sum_{i=1}^{N}\left(\xi_{Mi}-\bar{\xi_{M}}\right)}^{2}}$$
  where $\xi_{O}$ and $\xi_{M}$ are the observed data and the modeled data of the velocity-dip position, respectively, $\bar{\xi_{O}}$ and $\bar{\xi_{M}}$ are the average values of the observed data and the modeled data, respectively, and N is the total number of data points.
(2) The average relative error (RE) between the observed data points and the modeled data points is calculated by the following formula:
  (41) $$\mathrm{RE}=\frac{1}{N}\sum_{i=1}^{N}\frac{\left|\xi_{Oi}-\xi_{Mi}\right|}{\xi_{Oi}}*100(\%)$$
(3) The root mean square error (RMSE) between the observed data points and the modeled data points is calculated as follows:
  (42) $$\mathrm{RMSE}=\sqrt{\frac{1}{N}\sum_{i=1}^{N}{\left(\xi_{Oi}-\xi_{Mi}\right)}^{2}}$$
(4) The relative root mean square error (RRMSE) between the observed data points and the modeled data points is evaluated by the following formula:
  (43) $$\mathrm{RRMSE}=\sqrt{\frac{1}{N}\sum_{i=1}^{N}{\left(\frac{\xi_{Oi}-\xi_{Mi}}{\xi_{Oi}}\right)}^{2}}$$

It can be seen that the goodness of fit increases as *R*^2^ increases and RE, RMSE, and RRMSE decrease. Among these statistical parameters, the calculations of RE and RMSE values for the proposed Shannon entropy-based expression, existing conventional methods and experimental data were carried out in Kundu [9] and Kundu and Ghoshal [23], respectively.

### 3.3. Comparison Results

#### 3.3.1. For the Entire Cross Section of the Open Channel

**Figure 7:**
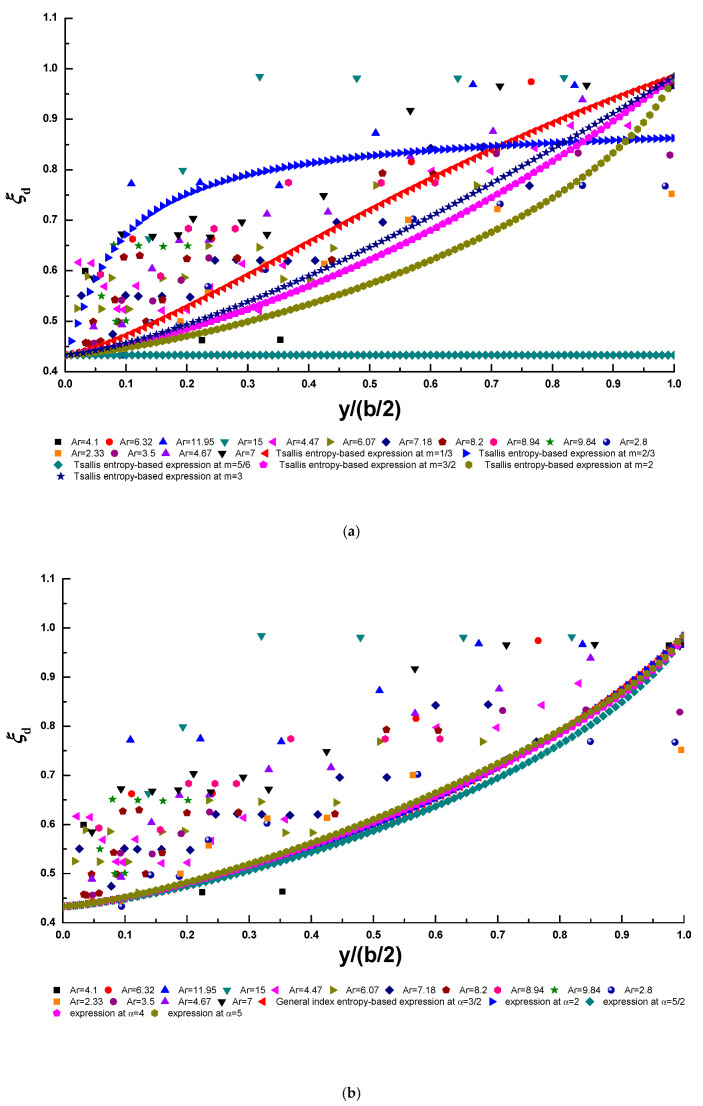

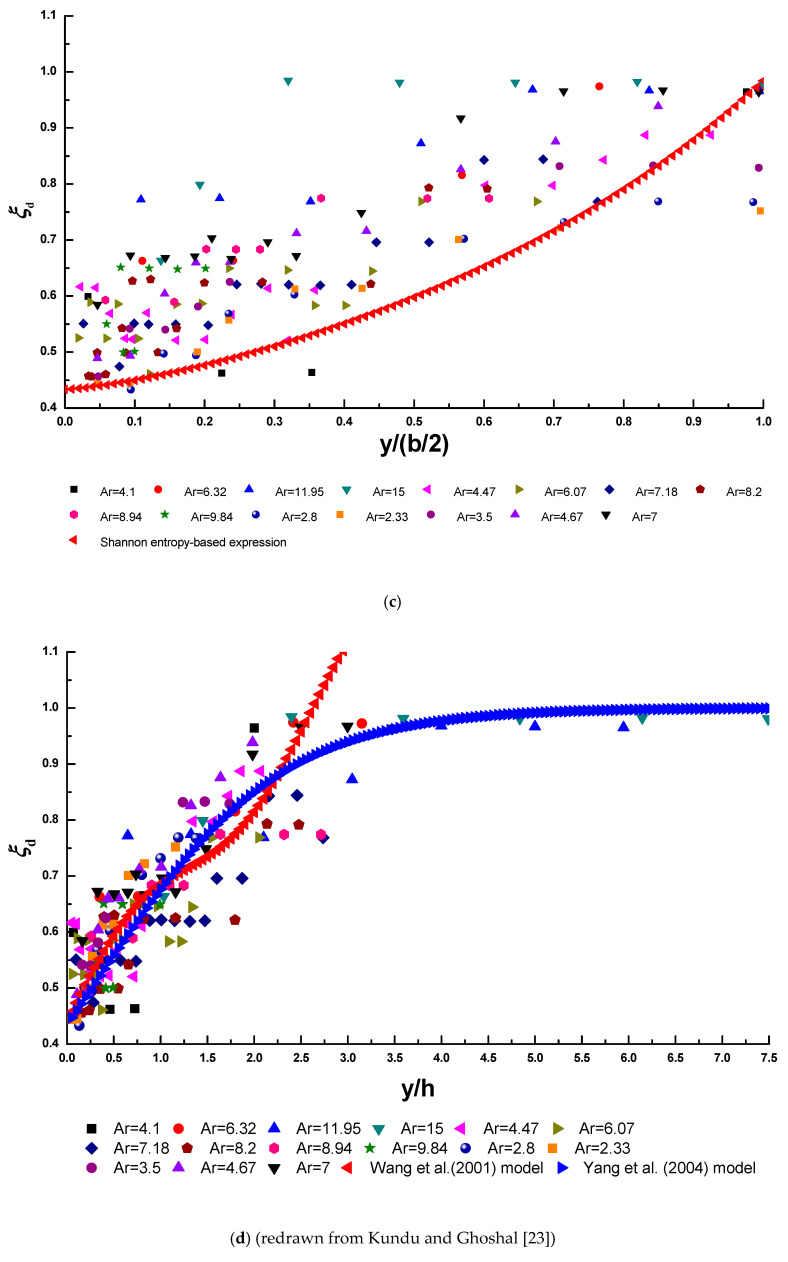
Comparison of the Tsallis entropy-based expression (**a**), the general index entropy-based expression (**b**), the Shannon entropy-based expression (**c**), and conventional deterministic models (**d**) for the velocity-dip position over the entire cross section of the open channel and the experimental data points.

To calculate the dimensionless entropy parameters $P_{T}$ and $P_{G}$, the non-linear equation systems (Equations (18) and (19) and Equations (27) and (28)) in this study need to be solved by means of the non-linear equation solver in MATLAB software. Here, the values of $\xi_{d\min}$, $\xi_{d\max}$ and $\bar{\xi_{d}}$ can be obtained from collected experimental data ($\xi_{d\min}$ = 0.4331, $\xi_{d\max}$ = 0.9842, and $\bar{\xi_{d}}$ = 0.6707), n = 1.361 is adopted as in Kundu and Ghoshal [23], and we simply choose various values of m and α in this study [41]: m = 1/3, 2/3, 5/6, 3/2, 2, 3; α = 3/2, 2, 5/2, 3, 7/2, 4, 5. For the Lagrange multiplier $\lambda_{S2}$ in the Shannon entropy-based expression (Equation (32)), the non-linear equation system (Equations (33) and (34)) is solved when $\xi_{d\min}$, $\xi_{d\max}$ and $\bar{\xi_{d}}$ are taken from collected experimental data. Figure 7a–d show the comparisons of the Tsallis entropy-based expression with various values of the entropy index m, the general index entropy-based expression with various values of the entropy index α, the Shannon entropy-based expression, and two conventional deterministic models (Equations (1) and (2)) and the experimental data, respectively. In Figure 7a, the Tsallis entropy index m is chosen to be 1/3, 2/3, 5/6, 3/2, 2, 3, and the general index entropy index α is chosen to be 3/2, 2, 5/2, 3, 7/2, 4, 5 in Figure 7b (the dimensionless entropy parameters $P_{G}$ cannot be obtained by solving the non-linear equation systems (Equations (27) and (28)) when α = 3 and 7/2, thus the cases of α = 3 and 7/2 are not presented in Figure 7b). Conventional deterministic models include the model of Wang et al. [13] (Equation (1)) and that of Yang et al. [14] (Equation (2)). A comparison among conventional deterministic models and experimental data (similar to that in Figure 7d) has already been presented in the work of Kundu and Ghoshal [23].

Table 1 presents the performance metrics of these models with respect to the experimental data by calculating the statistical parameter values given in Equations (40)–(43). The symbol **** denotes the highest *R*^2^ value and the lowest RE, RMSE, and RRMSE values for each case. The values shown in the fourth and fifth rows of this table are obtained at m = 1/3 and α = 5 respectively, at which the Tsallis entropy-based expression and the general index entropy-based expression best fit the experimental data points. It can be observed that the model of Yang et al. [14] can predict the lateral distribution of the velocity-dip position best among all of the proposed models. In addition, Tsallis entropy-based expression has the highest *R*^2^ value and the lowest RE, RMSE and RRMSE values among all the models, as indicated by the symbol *** in Table 1. This indicates that the Tsallis entropy-based expression could be a good addition to the existing deterministic model for predicting the lateral distribution of the velocity-dip position over the entire cross section of open channel flow.

#### 3.3.2. At the Centerline of the Open Channel

For a very narrow (or very deep) open rectangular channel, Hu and Hui [2] experimentally observed that the maximum flow velocity at the centerline of the open channel occurs in the middle of the water depth. This means that $\xi_{d\min}$ = 0.5. In contrast, for a very wide (or very shallow) open rectangular channel, the impact of the lateral boundaries of the open channel on the flow velocity profile at the centerline of the channel can be neglected, and consequently, the maximum flow velocity happens on the water surface (e.g., [2,15,16,20]). Thus, $\xi_{d\max}$ = 1. By substituting $\xi_{d\min}$ = 0.5 and $\xi_{d\max}$ = 1 into Equations (35), (37) and (39), it can be found that all of the entropy-based expressions satisfy two asymptotic boundary conditions, as proposed in Hu and Hui [2] and Guo [20], i.e., $\xi_{d}\to$0.5 as $A_{r}\to 0$ and $\xi_{d}\to$1 for $A_{r}\to \infty$. To estimate the entropy parameters $P_{T}$ and $P_{G}$, the non-linear equation systems (Equations (18) and (19) and Equations (27) and (28)) are solved by substituting $\xi_{d\min}$ = 0.5 and $\xi_{d\max}$ = 1, adopting the values of $\bar{\xi_{d}}$ from collected experimental data ($\bar{\xi_{d}}$ = 0.7718) and using some different values of m and α as follows [43]: m = 1/3, 2/3, 5/6, 3/2, 2, 3; α = 3/2, 2, 5/2, 3, 4, 5, 6. For the Shannon entropy-based expression (Equation (35)), the non-linear equation system (Equations (33) and (34)) is solved for the Lagrange multiplier $\lambda_{S2}$ when $\xi_{d\min}$, $\xi_{d\max}$ and $\bar{\xi_{d}}$ are given, n = 1.361, and $k_{1}$ = 0.0698 and $k_{2}$ = 1.8767, as presented in Kundu and Ghoshal [23] and Kundu [9].

**Figure 8:**
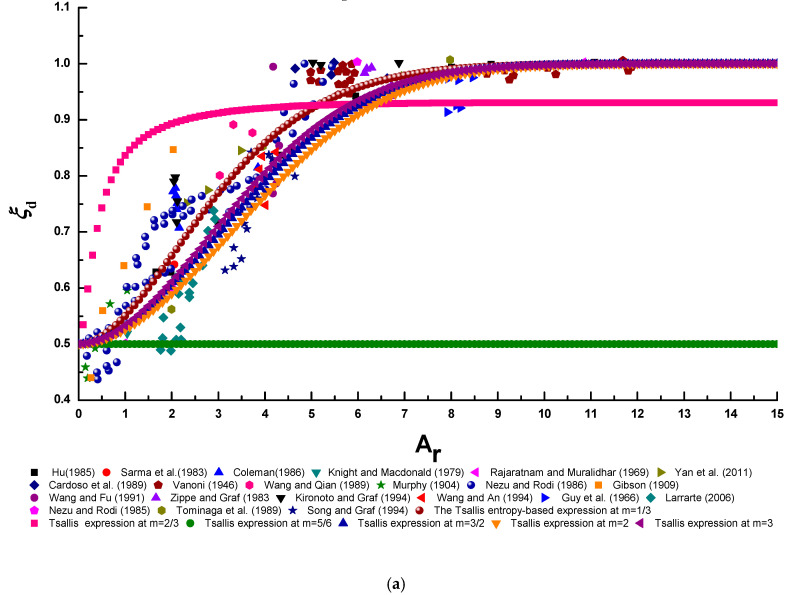

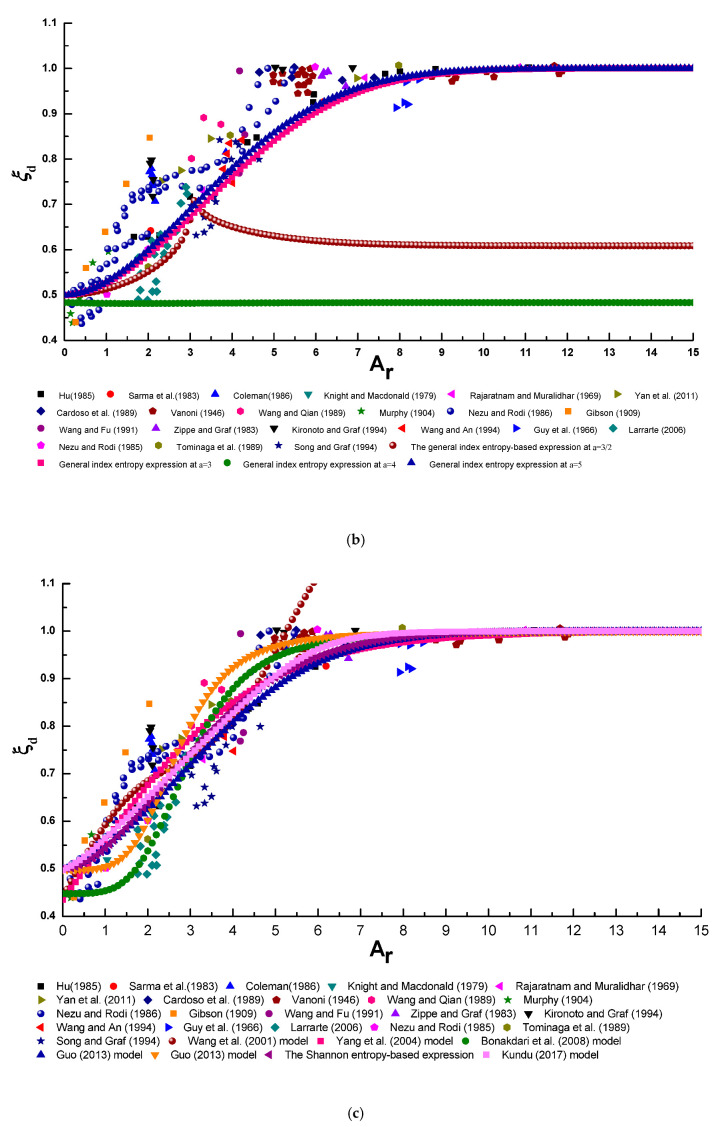
Comparison of the Tsallis entropy-based expression (**a**), the general index entropy-based expression (**b**), and the Shannon entropy-based expression and conventional deterministic models (**c**) for the velocity-dip position at the centerline of the open channel and the experimental datasets, respectively.

Figure 8a–c present a comparison of the Tsallis entropy-based expression with various values of the entropy index m, the general index entropy-based expression with various values of the entropy index α, and the Shannon entropy-based expression, six conventional deterministic models (Equations (3)–(8)) and the experimental data, respectively. In Figure 8a, the Tsallis entropy index m is set to 1/3, 2/3, 5/6, 3/2, 2, 3. The values of the general index entropy index α = 3/2, 3, 4, 5 are presented in Figure 8b, but α = 2,5/2, and 6 are not shown in this figure since, for the case of α= 2, they deviate from the experimental data to such an extent that they even lead to a negative value of the velocity-dip position, and for the cases of α = 5/2 and 6, the dimensionless entropy parameters $P_{G}$ cannot be obtained by solving the non-linear equation systems (Equations (27) and (28)). Conventional deterministic models include the models of Wang et al. [13] (Equation (3)), Yang et al. [14] (Equation (4)), Bonakdari et al. [19] (Equation (5)), Guo [20] (Equation (6)) and Pu [21] (Equation (7)). A comparison among the Shannon entropy-based expression, six conventional models except for Kundu [22] model and experimental data (Figure 8c) has already been presented in the work of Kundu [9]. A comparison among six conventional models and experimental data (Figure 8c) has already also been presented in the work of Kundu [22].

Table 2 shows the performance metrics of these models with respect to the experimental data by estimating the statistical parameter values given in Equations (40)–(43). The symbol **** denotes the highest *R*^2^ value and the lowest RE, RMSE, and RRMSE values for each case. The values shown in the eighth and ninth rows of this table are obtained at m = 1/3 and α = 5, respectively, at which the Tsallis entropy-based expression and the general index entropy-based expression best fit the experimental data points. It can be seen that the model of Kundu [22] has superior prediction accuracy for experimental data among all the conventional and entropy-based methods (the Shannon entropy-based expression has the same RRMSE value as the model of Kundu [22]). Except for the Kundu [22] model, the Shannon entropy-based expression can yield the highest *R*^2^ value and the lowest RE and RMSE values for experimental data, as shown by the symbol *** in Table 2. Furthermore, aside from the model of Kundu [22] and the Shannon entropy-based expression, Tsallis entropy-based expression has the highest *R*^2^ value and the lowest RMSE value, whereas the model of Yang et al. [14] and that of Guo [20] correspond to the lowest RE and RRMSE values among the other models, respectively, as shown by the symbol ** in Table 2. This shows the potential of entropy theory, particularly the Shannon entropy and the Tsallis entropy, to predict the velocity-dip position at the centerline of both narrow and wide open channels.

### 3.4. Physical Interpretation

**Figure 9:**
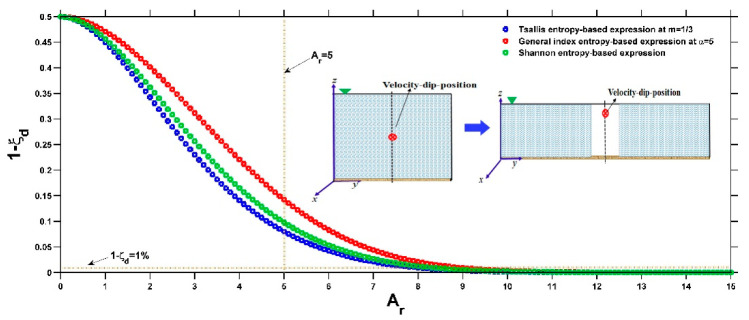
The impact of the lateral boundary of the open channel on the velocity-dip position at the centerline of the open channel as the aspect ratio of the channel varies based on the Tsallis, general index and Shannon entropy-based expressions with m = 1/3 and α = 5. In this figure, the insert is modified from Kundu [9].

For the velocity-dip position at the centerline of the open channel, the impact of the lateral boundary of the open channel on the position of the maximum streamwise velocity at the centerline of the channel could be characterized as $1-\xi_{d}$. Figure 9 shows the influence of the lateral boundary on the velocity-dip position at the centerline of the open channel with an increasing aspect ratio of the open channel based on the Tsallis entropy, general index entropy and Shannon entropy-based expressions (Equations (35), (37) and (39), respectively), with m = 1/3 and α = 5.

It can be observed that the impact of the lateral boundary on the velocity profile decays exponentially with the increasing width of the open channel (relative to the water depth), regardless of the type of entropy-based expression. For a narrow open channel with an aspect ratio $A_{r}$ < 5, the influence of the lateral boundary has an approximate range of 0.1 to 0.5, which shows that the impact of the lateral boundary on the velocity profile is not be negligible over the cross section of the channel. This point agrees with the analysis results of Kundu and Ghoshal [23]. When the open channel becomes wide, especially as $A_{r}$ ≥ 7, the influence of the lateral boundary is less than 3% for the Tsallis and Shannon entropy-based expressions, which indicates that the lateral boundary has a negligible effect on the velocity profile at the centerline of the open channel. These conclusions are consistent with the experimental results of Vanoni [58] and Nezu and Rodi [15]; in particular, when $A_{r}$ ≥ 8, $1-\xi_{d}$ is less than 1% for the Tsallis and Shannon entropy-based expressions, which suggests that the flow could be regarded as two-dimensional, in accordance with the results of Guo [20].

In the work of Kumbhakar and Ghoshal [59], the authors derived an analytical expression for the vertical distribution of longitudinal velocity in open channel flows based on the Renyi entropy. The CDF of longitudinal velocity was hypothesized to be the ratio of the flow depth at a point for which velocity is to be determined and the total water depth of the channel, meaning that the velocity increases monotonically from zero at the bottom of the channel to the maximum value at the water surface. According to the discussions mentioned above, it will be true only for open channel flows with a large aspect ratio (at least $A_{r}$ > 5). Therefore, the Renyi entropy-based one-dimensional velocity distribution formula derived by Kumbhakar and Ghoshal [59] could be applicative for a wide open channel flow (at least $A_{r}$ > 5).

## 4. Concluding Remarks

This study derived analytical expressions for the velocity-dip position over the entire cross section and at the centerline of the rectangular open channel by adopting probability methods based on the Tsallis and general index entropy theories. The derived Tsallis and general index entropy-based expressions had the same mathematical form as a function of the lateral distance from the sidewall of the channel or the aspect ratio of the open channel.

For the velocity-dip position over the entire cross section of the rectangular open channel, two existing conventional models and the Tsallis, general index, and Shannon entropy-based expressions are compared using fifteen experimental datasets from the literature. By performing an error analysis, it was found that the model of Yang et al. [14] predicts the lateral distribution of velocity-dip position the most effectively among all of the proposed models. With the exception of that model, the Tsallis entropy-based expression has the highest correlation coefficient value and the lowest relative error, root mean square error and relative root mean square error values among the other models. This indicates that the Tsallis entropy could be a good addition to existing deterministic models for predicting the lateral distribution of the velocity-dip position over the entire cross section of rectangular open channel flow.

Six existing conventional models and the Tsallis, general index, and Shannon entropy-based expressions are tested against twenty-one experimental datasets from the literature regarding the velocity-dip position at the centerline of the rectangular open channel. By performing an error analysis, it was found that the model of Kundu [22] has superior prediction accuracy with respect to the experimental data among all the models, and the Shannon entropy-based expression has the same RRMSE value as the model of Kundu [22]. With the exception of the model of Kundu [22] and the Shannon entropy-based expression, the Tsallis entropy-based expression has the highest correlation coefficient value and the lowest root mean square error value, whereas the model of Yang et al. [14] and that of Guo [20] correspond to the lowest relative error and relative root mean square error values among the other models, respectively. This shows the potential of entropy-based expressions, particularly the Shannon entropy and Tsallis entropy, to predict the velocity-dip position at the centerline of both narrow and wide rectangular open channels.

By means of the derived Tsallis and general index entropy-based expressions, it was also found that the lateral boundary has a noticeable impact on the velocity profile at the centerline of the rectangular open channel for a narrow open channel with an aspect ratio lower than 5. As the rectangular open channel becomes wide, particularly for the case in which aspect ratio is higher than 8, the influence of the lateral boundary on the flow velocity profile becomes negligible, and thus, two-dimensional flow occurs. These conclusions are consistent with previous experimental results.

## Figures and Tables

**Table 1:** Performance metrics of three kinds of entropy-based expressions and two existing deterministic models for twenty-one experimental datasets collected from the literature. The RE and RMSE values for the models of Wang et al. [13] and Yang et al. [14] were also calculated by Kundu and Ghoshal [23], and we calculate them again in this study, showing that these values are somewhat different.

Model Name	Prediction Accuracy			
	*R* ^2^	RE	RMSE	RRMSE
Wang et al. model [13]	0.6215	12.4208	0.1620	0.1878
Yang et al. model [14]	0.7908 ****	9.1990 ****	0.0743 ****	0.1157 ****
Tsallis entropy-based expression (m = 1/3)	0.6832 ***	12.1956 ***	0.1068 ***	0.1526 ***
General index entropy-based expression (α = 5)	0.6284	16.8727	0.1425	0.1961
Shannon entropy-based expression	0.6180	17.6436	0.1478	0.2035

**Table 2:** Performance metrics of three kinds of entropy-based expressions and six existing deterministic models for twenty-one experimental datasets collected from the literature. The RE and RMSE values for the six conventional models except for Kundu [22] model and the Shannon entropy-based expression were also calculated by Kundu [9], the RE and RMSE values for the six conventional models were also calculated by Kundu [22] and we calculate them again here, showing that they are different.

Model Name	Prediction Accuracy			
	*R* ^2^	RE	RMSE	RRMSE
Wang et al. model [13]	0.6971	13.4833	0.1768	0.1967
Yang et al. model [14]	0.8466	7.6692 **	0.0681	0.1053
Bonakdari et al. model [19]	0.8125	10.0856	0.0979	0.1385
Guo model [20]	0.8470	7.7653	0.0790	0.0994 **
Pu model [21]	0.7942	9.5668	0.0882	0.1256
Kundu [22] model	0.8601 ****	7.2453 ****	0.0647 ****	0.0983 ****
Tsallis entropy-based expression (m = 1/3)	0.8493 **	7.6886	0.0675 **	0.1035
General index entropy-based expression (α = 5)	0.8392	8.4620	0.0807	0.1069
Shannon entropy-based expression	0.8520 ***	7.4116 ***	0.0668 ***	0.0983 ****
